# Anticonvulsant effects of mefloquine on generalized tonic-clonic seizures induced by two acute models in rats

**DOI:** 10.1186/s12868-015-0145-7

**Published:** 2015-03-01

**Authors:** Javier Franco-Pérez, Paola Ballesteros-Zebadúa, Joaquín Manjarrez-Marmolejo

**Affiliations:** Laboratory of Physiology of Reticular Formation, National Institute of Neurology and Neurosurgery, M.V.S, Insurgentes Sur 3877, Col. La Fama, C.P. 14269 Mexico, DF Mexico; Laboratory of Medical Physics, National Institute of Neurology and Neurosurgery, M.V.S, Mexico, DF Mexico

**Keywords:** Gap junction, Maximal electroshock, Mefloquine, Pentylenetetrazole, Seizures

## Abstract

**Background:**

Mefloquine can cross the blood–brain barrier and block the gap junction intercellular communication in the brain. Enhanced electrical coupling mediated by gap junctions is an underlying mechanism involved in the generation and maintenance of seizures. For this reason, the aim of this study was to analyze the effects of the systemic administration of mefloquine on tonic-clonic seizures induced by two acute models such as pentylenetetrazole and maximal electroshock.

**Results:**

All the control rats presented generalized tonic-clonic seizures after the administration of pentylenetetrazole. However, the incidence of seizures induced by pentylenetetrazole significantly decreased in the groups administered systematically with 40 and 80 mg/kg of mefloquine. In the control group, none of the rats survived after the generalized tonic-clonic seizures induced by pentylenetetrazole, but survival was improved by mefloquine. Besides, mefloquine significantly modified the total spectral power as well as the duration, amplitude and frequency of the epileptiform activity induced by pentylenetetrazole. For the maximal electroshock model, mefloquine did not change the occurrence of tonic hindlimb extension. However, this gap junction blocker significantly decreased the duration of the tonic hindlimb extension induced by the acute electroshock.

**Conclusions:**

These data suggest that mefloquine at low doses might be eliciting some anticonvulsant effects when is systemically administered to rats.

## Background

Mefloquine is a quinoline derivative available since 1985 as an antimalarial agent and is still considered highly effective for malaria chemoprophylaxis in populations with particular characteristics as pregnant women, children, and travelers [1,2]. It has been showed that mefloquine can quickly cross the blood–brain barrier and remain in the brain even for more than 24 hours [3-5].

*In vitro* studies established that mefloquine blocks certain types of gap junction channels and consequently modifies the gap junctional coupling between both cortical and hippocampal neurons [6-8]. The gap junction channels are expressed in neurons and glial cells providing cytoplasmic continuity and direct communication between neighboring cells. These transmembranal channels contribute to the fast exchange of ions and some small molecules thus allowing the electrical coupling and the neuronal hypersynchronic activity [9-11].

The neuronal hypersynchronic activity drives to convulsive events and hence is a hallmark of epilepsy. For this reason, it has emerged a hypothesis proposing that enhanced electrical coupling mediated by gap junctions is an underlying mechanism involved in the pathophysiological generation of seizure activity [12,13].

Nowadays, there are an increasing number of attempts trying to link seizures and gap junctional coupling. As a result, some studies have analyzed the effects produced by different gap junction blockers on seizures models, and diverse results have been described. *In vivo* studies have observed that the administration of quinine, a mefloquine-related compound, has anticonvulsant properties, even when administered by diverse routes and evaluated in different seizure models [14-17]. By contrast, paradoxically it has been proposed that quinine and also mefloquine show some excitatory effects increasing the frequency of seizure-like events in rat cortical slices [7]. Although mefloquine seems to be more specific than other gap junction blockers [18], its effects on acute seizure models are underrepresented.

In order to contribute clarifying this matter, the aim of this study was to analyze the effects of the systemic administration of mefloquine on generalized tonic-clonic seizures (GTCS) and epileptiform activity induced by pentylenetetrazole (PTZ) as well as on tonic hindlimb extension (THLE) induced by maximal electroshock (MES). The present results suggest that mefloquine could be eliciting some protection against the seizures triggered by two acute models.

## Results

### PTZ model

Previous to analyze the effects of mefloquine, we compared two control groups (DMSO 50% + PTZ vs. saline solution + PTZ) on the behavior and epileptiform activity induced by PTZ. We did not find significant differences between these groups (data not shown) and, for this reason; we decided only to include the vehicle group (DMSO 50%) as the unique control.

**Figure 1:**
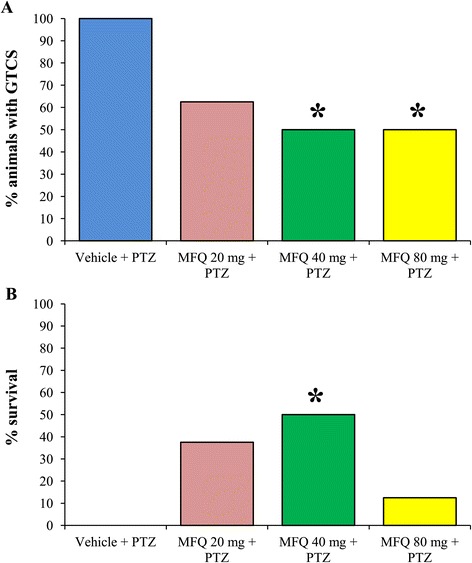
**Mefloquine decreases the incidence of generalized tonic-clonic seizures and increases the percentage of survival. A)** The intraperitoneal administration of mefloquine (MFQ) significantly decreases the incidence of generalized tonic-clonic seizures (GTCS) and **B)** significantly increases the percentage of survival after the administration of pentylenetetrazole (PTZ). The level of significance (*p < 0.05) was determined by independent Fisher’s exact probability tests comparing each experimental group versus control vehicle group. Vehicle + PTZ (n = 8), MFQ 20 mg/kg + PTZ (n = 8), MFQ 40 mg/kg + PTZ (n = 8), MFQ 80 mg/kg + PTZ (n = 8).

**Figure 2:**
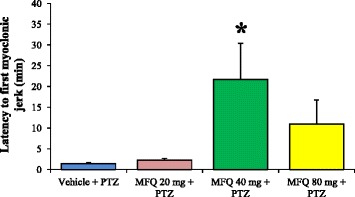
**Influence of mefloquine on the latency to the first myoclonic jerk induced by pentylenetetrazole.** The latency to the first myoclonic jerk was significantly increased after the administration of mefloquine (MFQ) 40 mg/kg. Values are expressed in minutes as mean ± SEM. The level of significance (*p < 0.05) was determined by nonparametric Kruskal-Wallis analysis followed by Dunnet test. Vehicle + PTZ (n = 8), MFQ 20 mg/kg + PTZ (n = 8), MFQ 40 mg/kg + PTZ (n = 8), MFQ 80 mg/kg + PTZ (n = 8).

In the control group, the 100% of the animals administered with vehicle plus PTZ presented GTCS. These GTCS were characterized by a first clonic phase with myoclonus of the anterior limbs followed by a tonic phase characterized by hindlimbs extension and finally a second clonic phase with myoclonus of the posterior limbs. The administration of mefloquine induced a response although it was not clearly dose-related. It was observed that the incidence of GTCS decreased to 62.5% in the group treated with 20 mg/kg; however, this change was not statistically significant. Meanwhile, in both groups administered with 40 and 80 mg/kg of mefloquine there was a significant reduction (p < 0.05) of 50% in the incidence of GTCS (Figure 1A). In this study, we observed that in the control group, none rat survived after the expression of the GTCS induced by PTZ. This parameter was modified by mefloquine (p < 0.05). As a result, it was observed the 37.5, 50 and 12.5% of survival with the doses of 20, 40 and 80 mg/kg of mefloquine respectively (Figure 1B). The intraperitoneal administration of PTZ 70 mg/kg quickly induced visible behavioral changes in all rats. The first behavioral sign presented after PTZ administration was the myoclonic jerk characterized by an intense shaking of the whole body. In the control group, this behavioral parameter was shown 1.4 ± 0.2 minutes after PTZ administration. However, mefloquine delayed the appearance of this behavioral parameter because the first myoclonic jerk was shown 2.2 ± 0.4 (20 mg/kg), 21.6 ± 8.7 (40 mg/kg) (p < 0.05) and 10.9 ± 5.8 minutes (80 mg/kg) after the administration of PTZ (Figure 2).

**Figure 3:**
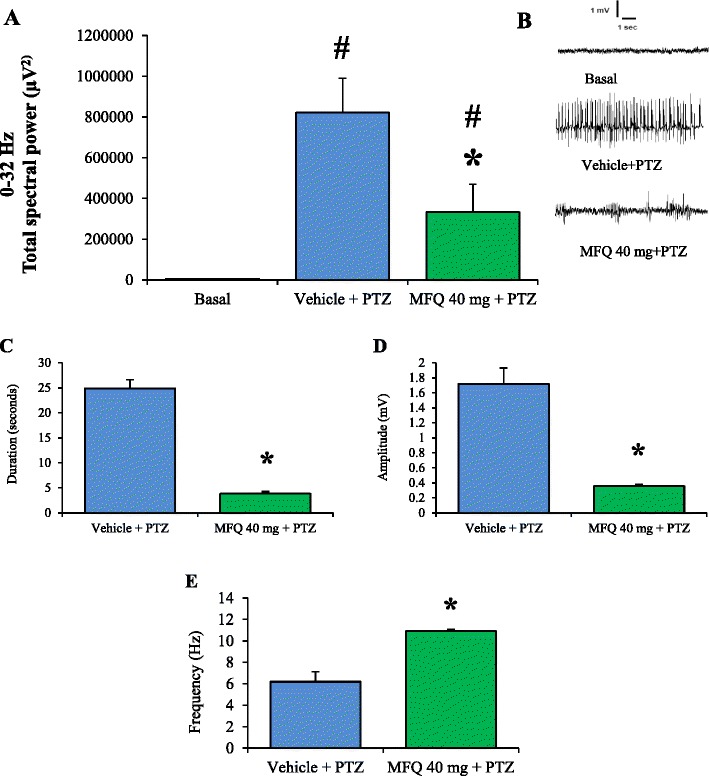
**Mefloquine significantly modifies the total spectral power of the EEG and some parameters of the epileptiform activity induced by pentylenetetrazole.** Mefloquine (MFQ) (40 mg/kg) significantly decreased the total spectral power and modified the epileptiform activity in rats administered with pentylenetetrazole (PTZ). **A)** Total spectral power acquired in the frequency ranges of 0–32 Hz; error bars represent SEM. **B)** Representative EEG traces obtained in 10-seconds epochs during basal recording and post-administration of vehicle or MFQ and PTZ. **C)** Duration, **D)** Amplitude and **E)** Frequency of the epileptiform activity induced by PTZ. MFQ significantly modified these parameters. *p < 0.05 statistically significant compared with the control group (vehicle + PTZ). #p < 0.05 statistically significant compared with the basal recording. The total spectral power was analyzed using a Nonparametric Kruskal-Wallis analysis followed by Student Newman Keuls test. The duration, amplitude, and frequency were compared with a Mann–Whitney rank sum test. Vehicle + PTZ (n = 8), MFQ 40 mg/kg + PTZ (n = 8).

The dose of 40 mg/kg was the only one that significantly modified all the behavioral parameters evaluated after the administration of PTZ. For this reason, 40 mg/kg of mefloquine was selected to analyze the total spectral power of the EEG, as well as the duration, amplitude and frequency of the epileptiform activity induced by PTZ. As a result, it was found that mefloquine significantly decreased (p < 0.05) the total spectral power of the EEG compared with the control group (Figure 3A, B). Similarly, mefloquine significantly modified the epileptiform activity decreasing the duration and amplitude and increasing the frequency of the epileptiform activity induced by PTZ (Figure 3C-E).

**Figure 4:**
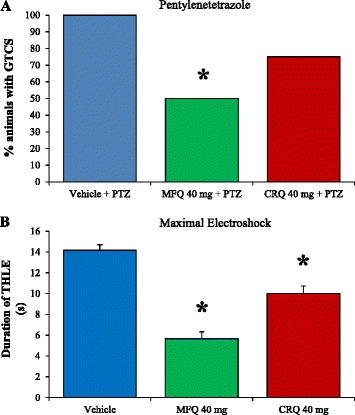
**Effects of chloroquine and mefloquine on the generalized seizures induced by pentylenetetrazole and maximal electroshock. A)** Chloroquine (CRQ) did not protect against the generalized tonic-clonic seizures (GTCS) induced by pentylenetetrazole (PTZ). The level of significance (*p < 0.05) was determined by independent Fisher’s exact probability test comparing each experimental group versus control vehicle group. Vehicle + PTZ (n = 8), MFQ 40 mg/kg + PTZ (n = 8), CRQ 40 mg/kg + PTZ (n = 8). **B)** CRQ did not modify the incidence of tonic hindlimb extension (THLE) induced by maximal electroshock (MES). However, this drug decreased the duration of THLE but in minor proportion that a similar dose of mefloquine. The level of significance (*p < 0.05) was determined by nonparametric Kruskal-Wallis analysis followed by Dunnet test. Vehicle (n = 6), MFQ 40 mg/kg (n = 6), CRQ 40 mg/kg (n = 6).

In order to relate the effects of mefloquine with the blockage of gap junctions, we conducted an additional experiment using chloroquine. This compound is chemically related to mefloquine but does not block gap junctions. Interestingly, a similar dose of chloroquine (40 mg/kg), did not significantly modify the incidence of GTCS induced by PTZ (Figure 4A).

### MES model

The MES model induced THLE in all the rats evaluated including those administered with different doses of mefloquine. Although the THLE was the only parameter measured, at the end of this tonic phase, we also observed in all rats a clonic phase characterized by paddling movements of the limbs and shaking of the body.

**Figure 5:**
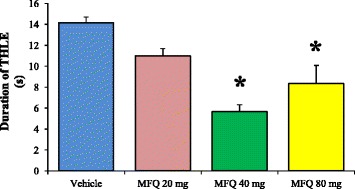
**Duration of tonic hindlimb extension induced by maximal electroshock after the administration of mefloquine.** The duration of tonic hindlimb extension (THLE) was significantly reduced after the administration of different doses of mefloquine (MFQ). Values are expressed in seconds as mean ± SEM. The level of significance (*p < 0.05) was determined by nonparametric Kruskal-Wallis analysis followed by Dunnet test. Vehicle (n = 6), MFQ 20 mg/kg (n = 6), MFQ 40 mg/kg (n = 6), MFQ 80 mg/kg (n = 6).

The control group administered with vehicle exhibited THLE with a mean duration of 14.1 ± 0.54 seconds. Interestingly, the evaluated doses of mefloquine induced a decrease of the THLE duration. This decrease was statically significant (p < 0.05) only in the groups administered with 40 mg/kg (5.6 ± 0.66 seconds) and 80 mg/kg (8.3 ± 1.74 seconds) of mefloquine (Figure 5).

When analyzed the effects of chloroquine (40 mg/kg), we also observed that this antimalarial drug did not protect against the THLE induced by MES. However, the chloroquine significantly decreased the duration of THLE (10 ± 0.70 seconds) but in minor proportion that a similar dose of mefloquine (40 mg/kg) (5.6 ± 0.66 seconds) (Figure 4B).

## Discussion

Consistently, it has been assumed that gap junction blockers have certain anticonvulsant properties. Quinine, another antimalarial drug chemically related to mefloquine, blocks gap junction channels similar to those blocked by mefloquine [6,19]. Comparable to our results, others authors have demonstrated that the systemic administration of quinine 40–60 mg/kg, significantly decrease the duration of seizures induced by PTZ [15]. The present results suggest that a single dose (40 mg/kg) of mefloquine protect against the GTCS induced by PTZ and also reduce the duration of THLE induced by MES.

Despite to be clinically tested as an antimalarial drug [1,2] and to be proposed as a specific gap junction blocker [6], mefloquine has not been adequately evaluated as a possible anticonvulsant. Probably, this fact is related to controversial reports that have associated mefloquine with proconvulsant effects. Voss and collaborators [7], described that *in vitro* mefloquine induced an increase in the spontaneous local field potential activity denominated as seizure-like activity. Another study showed that in mice, doses as high as 137.5 mg/kg of mefloquine elicited spontaneous tonic seizures probably modulated by GABAergic mechanisms [20].

The maximal dose of mefloquine used in our study was 80 mg/kg, and this treatment decreased 50% the incidence of GTCS induced by PTZ and also reduced the duration of THLE induced by MES. Interestingly, others authors have reported that rats administered with higher doses of mefloquine exhibited excitatory behaviors such as wild running [20]. However, our observations indicate that rats administered with mefloquine, even at the maximal dose used, showed a sedation-like state characterized by decrease in spontaneous locomotor activity and seemingly sleepiness. Similarly, it has been observed that mefloquine (50 mg/kg) robustly suppressed the tremor in a mouse model of essential tremor [21]. This finding suggests that mefloquine could be inhibiting the hypersynchronized firing of neurons in the anatomical substrates involved in the tremor and consequently induces a reduction of motor activity.

Interestingly, mefloquine reduced the incidence and amplitude of the epileptiform activity induced by PTZ. Some hypotheses suggest that gap junctions are related to the neuronal hypersynchronic activity that drives the convulsive events [12,13]. In accordance with this proposal, some studies have showed that in cerebral pathologies characterized by seizures there is a cellular hyperexcitability related to increased gap junctional intercellular coupling in cortical pyramidal neurons [22]. Moreover, comparable with our results, it has been reported that mefloquine reduced the incidence, amplitude and duration of recurrent epileptiform discharges in hippocampal slices bathed with bicuculline, a GABA_A_ receptor blocker similar to the PTZ [23]. Given that PTZ induces neuronal hypersynchonic activity and mefloquine decreased the amplitude fluctuations, our results suggest that mefloquine could be exerting its anticonvulsant effects interfering with the synchronization of the neuronal activity.

Additionally, in order to relate the effects of mefloquine with the blockage of gap junctions, we used chloroquine, an antimalarial drug chemically related to mefloquine but without effects on gap junctional intercellular communication [21]. Interestingly, chloroquine showed only a slight modification of the incidence of GTCS induced by PTZ as well as on the duration of the THLE induced by MES. However, these modifications were of minor magnitude that those observed with mefloquine. Given the differences found between mefloquine and chloroquine, we suggest that the blockage of gap junctions could be a mechanism involved in the anticonvulsant effects of mefloquine.

The clinical use of mefloquine has been criticized because of the neurological side effects observed such as nausea, dizziness, sleep disturbances, anxiety, psychosis, and convulsions [24]. However, animal studies have suggested that neurological effects induced by mefloquine are dose-dependent and that the threshold dose for the appearance of undesired side effects is around 187 mg/kg [25]. In our study, the protective treatment against the GTCS induced by PTZ and the THLE induced by MES was 40 mg/kg. For this reason, we can propose that 40 mg/kg of mefloquine is a safe dose and free of secondary effects. As a result, seems obvious that the beneficial impact of mefloquine may be inverted with high doses. Since low doses could be anticonvulsant, high doses could be proconvulsant and even neurotoxic.

Mefloquine shows low solubility in typical solvents such as water or saline solution, for this reason, we used DMSO as vehicle. Because the chemical properties, DMSO is a solvent frequently utilized in a great variety of biological studies [26]. Although the utilization of this solvent has been criticized, DMSO has been described as a useful vehicle for diverse seizure studies. Specifically, it has been showed that the direct intracerebroventricular administration of DMSO 100% did not interfere with the epileptiform activity induced by proconvulsant drugs [27]. Even, we did not find significant differences when comparing two control groups (DMSO 50% + PTZ vs. saline solution + PTZ) on the behavior and epileptiform activity induced by PTZ.

Overall, it has been assumed that mefloquine elicits a specific blockage of gap junctions [6-8] and, as a result, inhibit the hypersynchronized firing of neurons in some neuroanatomical substrates. However, some authors have proposed that mefloquine also modify the activity of chemical synaptic neurotransmission. Specifically, Zhou and collaborators [28] established that, *in vitro*, mefloquine enhances GABA release onto midbrain dopaminergic neurons. Probably, after administration of mefloquine, this enhanced release could be present in the brain and, as a result, contribute to the anticonvulsant effects observed in this work. However, although these findings give us an approach to the problem, more studies are necessary to determine if the anticonvulsant effects showed by mefloquine are mediated mainly by gap junctions or by a set of mechanisms acting in concert.

## Conclusions

We found that systemic administration of mefloquine produces some anticonvulsant effects in two acute seizure models such as PTZ and MES. Despite the controversy about the neurotoxic and proconvulsant effects induced by mefloquine at high doses, we propose that mefloquine at low doses could be useful to evaluate anticonvulsant effects in seizure experimental models.

## Methods

### Animals

Seventy male Wistar rats (270–300 g) were maintained under controlled conditions (24.4°C; 7:00–19:00 light, 19:00–7:00 darkness) as well as with free access to food and water. All animals were treated according to regulations specified by the Bioethical Committee of the National Institute of Neurology and Neurosurgery M.V.S. and according to the technical specifications of the Mexican Standard for the production, care and use of laboratory animals (NOM-062-ZOO-1999). Additionally, the Guide for the Care and Use of Laboratory Animals (NIH Guide) was revised and used as guidelines.

### Groups

For the PTZ model, the rats were randomly divided into five groups of eight animals each for a total of forty rats. The control group was injected intraperitoneally (i.p.) with the vehicle (saline solution plus dimethyl sulfoxide (DMSO), 1:1). Three experimental groups were given with mefloquine dissolved in the vehicle at the doses of 20, 40 and 80 mg/kg. 30 minutes after mefloquine, the rats were administered with PTZ (70 mg/kg, i.p.). One additional experimental group was given with chloroquine (40 mg/kg) dissolved in the vehicle. 30 minutes after mefloquine, the rats were administered with PTZ (70 mg/kg, i.p.).

For the MES model, we used thirty rats randomly divided into five groups of six animals each. The control group was injected i.p. with vehicle (saline solution plus DMSO, 1:1). Three experimental groups were administered with mefloquine dissolved in the vehicle at the doses of 20, 40 and 80 mg/kg 30 minutes before the maximal electroshock. One additional experimental group was given with chloroquine (40 mg/kg) dissolved in vehicle 30 minutes before the maximal electroshock.

### Implantation of electrodes for electroencephalographic (EEG) recordings

Animals were anesthetized i.p. with a mixture of ketamine (Pisa, Mexico) (75 mg/kg) and xylazine (Pisa, Mexico) (10 mg/kg). In accordance with some guidelines for the use of anesthetics, this mixture is suitable for restraint procedures [29]; however, the cardiac and respiratory rates always were observed to ensure the correct anesthesia of the animals. As previously described [30], two electrodes made of stainless-steel Teflon-coated wires (A-M Systems Inc. Carlsborg, WA) with uncoated tips were deeply implanted in the motor cortex (1.2 mm anterior to Bregma, 2.5 mm lateral to the midline, 1.5 mm below the surface of the skull) for EEG recordings. One more electrode implanted above the cerebellum was used as a reference (11.9 mm posterior to Bregma, 3.0 mm lateral to the midline, 2.0 mm below the surface of the skull). The electrodes were fixed to the skull with anchor screws and finally all the set was secured with dental cement. After surgery, the animals were allowed a week of recovery in their home-cage at the vivarium with controlled conditions.

### Drugs

Mefloquine hydrochloride and chloroquine diphosphate salt (Sigma-Aldrich. St Louis, MO) were dissolved in a solution of DMSO 50% (Sigma-Aldrich. St Louis, MO) prepared with saline solution. PTZ was dissolved in saline solution and administered with a dose of 70 mg/kg. All the solutions were made the same day of the experiment guarantying cleanliness and avoiding degradation. All the drugs were administered i.p. in a volume of 5 ml/kg. In accordance with Turner and collaborators [31]; in rats, the maximum amount to administrate for i.p route is 10 ml/kg. For this reason, 5 ml/kg is an ideal volume to facilitate the absorption of substances given i.p.

### PTZ model

Rats were connected to an amplifier model BE light (EBNeuro®. Firenze, Italy) by means of flexible cables to allow the free movement in a Plexiglass cage. The video-EEG was recording by means of Galileo NT software (EBNeuro®. Firenze, Italy). Before any administration, a basal EEG recording was carried out for approximately 10 minutes. At the end of basal recording, the vehicle or mefloquine were administered and after 30 minutes of drugs administration, the animals were injected with PTZ. Mefloquine quickly reaches the brain, and maximal brain concentrations have been observed after 30 minutes of i.p. administration [3-5]. At the final of the experiment, the video-EEG recordings were stored on the hard drive of a computer for the off-line analysis. Using the video-EEG recordings we observed and analyzed the behavioral and EEG signals associated with the GTCS induced by PTZ. The GTCS were behaviorally characterized by a first clonic phase with myoclonus of the anterior limbs followed by a tonic phase characterized by hindlimbs extension and finally a second clonic phase with myoclonus of the posterior limbs. In the EEG, the GTCS were characterized by burst of activity highly synchronized with notable voltage fluctuations and persistent for at least 2 seconds. Based on these behavioral and EEG characteristics, we determined the incidence percentages of GTCS and survival as well as the latencies to the first myoclonic jerk induced by PTZ.

### Analysis of total power spectral and other parameters of the epileptiform activity

We used a modification of the method previously described by Markovic and collaborators [32]. Using the Galileo NT software (EBNeuro®. Firenze, Italy), all EEG signals were filtered with a low-pass at 0.3 Hz and a high-pass at 70 Hz. The probable external noise was avoided by means of the 60 Hz notch filter. During rat wakefulness, 10-s epochs of the EEG recordings were extracted for the following analysis. The selection of 10-s epochs allowed includes EEG segments without artifacts due to cable adjustments or external noise. In addition, these EEG segments contained the epileptiform activity that has been defined as highly synchronized bursting activity with clear trains of voltage fluctuations and persistent for at least 2 seconds. This method has been recently used by others authors [32-34]. Afterward, the EEG recordings were subjected to an automated analysis based on the fast Fourier transform method in order to estimate the total spectral power (μV^2^) of the EEG signal. Besides, it was determined the duration (seconds), amplitude mean (mV) and frequency mean (Hz) of the epileptiform activity induced by PTZ. These analysis were carried out limiting the frequency range until 32 Hz because it has been demonstrated that PTZ induces epileptiform activity characterized by voltage fluctuations mainly at low frequencies (1–16 Hz) and with minimal fluctuations at high frequencies (>30 Hz) [33].

### MES model

A previous MES protocol [35] was modified because of the limitations of the maximal current output (100 mA) of our equipment (Hugo Basile, model 57800–001). In accordance with previous tests in our laboratory, we established the optimal parameters to induce THLE in rats: frequency 60 Hz, pulse width 0.6 ms, shock duration 0.6 s and current 90 mA. The vehicle or mefloquine were administered, and 30 minutes after the animals were placed in a Plexiglas cage to apply a single stimulation by means of ear clips. Because MES protocol is a behavioral model, the criterion for the occurrence of seizure activity was the THLE. Two experimenters observed the animal behavior in order to quantify the occurrence and duration of THLE. The duration of THLE was determined as the total time during which the rat presented maximal extension of the anterior and posterior limbs and when the body becomes outstretched 180° to the plane of the body axis. We did not perform EEG recordings to the rats designated to the MES model because the current stimulation *per se* can induce significant artifacts in the EEG recordings that could be confused with the epileptiform activity.

### Statistical analysis

Fisher’s exact probability tests (p < 0.05 significance level) were used to compare the incidence of GTCS and percentage of survival after administration of PTZ. We used independent Fisher’s exact tests to compare each experimental group against the control vehicle group; these comparisons have been previously used [30,32]. Also, Kruskal-Wallis test followed by either Dunnett’s or Dunn’s tests were used to determine the statistical significance (p < 0.05) of the latency to the first myoclonic jerk, the total spectral power, as well as to analyze the duration of THLE. The duration, amplitude and frequency of the epileptiform activity were analyzed with a Mann–Whitney rank sum test.

## Abbreviations

DMSO: Dimethyl sulfoxide
EEG: Electroencephalographic recordings
GTCS: Generalized tonic-clonic seizures
i.p: Intraperitoneal
MES: Maximal electroshock
MFQ: Mefloquine
PTZ: Pentylenetetrazole
THLE: Tonic hindlimb extension
